# Nationwide Real‐World Modeling of Surgical Outcomes in Elderly Patients: Incorporating Geriatric‐Specific Risk Factors Into Prediction of Mortality and Morbidity

**DOI:** 10.1002/ags3.70164

**Published:** 2026-01-11

**Authors:** Naoya Sato, Hiraku Kumamaru, Mitsukazu Gotoh, Yoshihiro Kakeji, Yuko Kitagawa, Yasuyuki Seto, Hiromi Rakugi, Masahiro Akishita, Kazue Nakajima, Arata Takahashi, Hiroaki Miyata, Shigeru Marubashi

**Affiliations:** ^1^ Department of Hepato–Biliary–Pancreatic and Transplant Surgery Fukushima Medical University Fukushima Japan; ^2^ Department of Healthcare Quality Assessment, Graduate School of Medicine The University of Tokyo Tokyo Japan; ^3^ Department of Health Data Science Yokohama City University Graduate School of Data Science Yokohama Japan; ^4^ Osaka General Medical Center Osaka Japan; ^5^ Division of Gastrointestinal Surgery, Department of Surgery Kobe University Graduate School of Medicine Kobe Japan; ^6^ Department of Surgery Keio University School of Medicine Tokyo Japan; ^7^ Department of Gastrointestinal Surgery, Graduate School of Medicine The University of Tokyo Tokyo Japan; ^8^ Department of Geriatric and General Medicine, Graduate School of Medicine Osaka University Osaka Japan; ^9^ Tokyo Metropolitan Institute for Geriatrics and Gerontology Tokyo Japan; ^10^ Department of Clinical Quality Management Osaka University Hospital Osaka Japan; ^11^ Department of Health Policy and Management Keio University School of Medicine Tokyo Japan

**Keywords:** comprehensive geriatric assessment, geriatric risk model, geriatric surgery

## Abstract

**Aim:**

As the aging population grows, it is crucial to evaluate the quality of surgical care considering geriatric‐specific factors and outcomes. The aim of this study was to develop and validate prediction models for mortality and morbidity in patients aged 65 and older by using a large‐scale, nationwide, real‐world dataset to enable robust and generalizable modeling and to evaluate the contribution of geriatric‐specific risk factors to the model.

**Methods:**

Data from the National Clinical Database in 2021, incorporating 22 geriatric‐specific variables, was used to develop prediction models for 30‐day mortality and major complications using 70% of the dataset (development cohort), with validation on the remaining 30% (validation cohort).

**Results:**

A total of 64 868 cases from gastroenterological surgeries were analyzed. In this geriatric cohort, the 30‐day mortality rate was 1.8%, and the major complication rate was 11.3%. The prediction models demonstrated strong discrimination and calibration (HL = 0.198 and c‐statistics = 0.84 for mortality, HL = 0.0003 and c‐statistics = 0.68 for morbidity). The mortality prediction model identified three significant geriatric‐specific factors as independent predictors: surrogate consent (odds ratio [OR] 1.91; 95% confidence interval [CI] 1.55–2.35), depression (OR 1.68; 95% CI 1.02–2.75), and hospitalization from outside the home (OR 1.24; 95% CI 1.01–1.54), along with age (75–79 years vs. 65–69 years, OR 1.62; 95% CI 1.20–2.19).

**Conclusions:**

Incorporating geriatric‐specific factors along with age identified clinically relevant predictors of mortality and morbidity, reinforcing the importance of geriatric assessment in elderly surgical patients.

## Graphical abstract

A reliable and effective risk prediction model for mortality and morbidity in elderly patients undergoing gastroenterological surgeries was developed and validated. Geriatric‐specific risk factors, including the newly added variables in the NCD registry, along with age, were identified as significant contributors to the model.

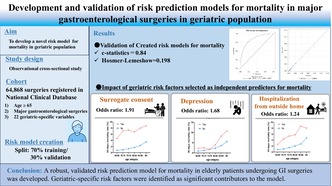

## 1 Introduction

The global population is aging, with a growing number of individuals over the age of 65 [1]. In Japan, the number of geriatric surgeries is also increasing [2]. This demographic shift heightens the demand for quality surgical care, as older populations generally require more healthcare due to physical decline, cognitive impairments, and social challenges [3, 4]. Given the higher risk of adverse surgical outcomes, there is a growing need for comprehensive geriatric assessments to identify vulnerabilities [5]. These assessments help evaluate a patient's ability to tolerate surgery from various perspectives.

Evaluating the quality of surgical care for elderly patients requires a comprehensive clinical database that includes data on surgical and geriatric outcomes, such as mortality, morbidity, and physical function decline, covering an entire nation or region. In Japan, the National Clinical Database (NCD), established in 2010, includes over 95% of all surgical procedures, collecting preoperative risk factors and tracking adverse outcomes for up to 90 days postoperatively. The accuracy of NCD data has been ensured through systematic audits initiated by the Japanese Society of Gastroenterological Surgery (JSGS) database committee in 2016 [6]. The use of the NCD registry plays a crucial role in assessing the quality of surgical care for elderly patients in Japan, a country experiencing rapid aging. In response to the growing focus on geriatric surgery, the American College of Surgeons (ASC) launched the Geriatric Surgery Pilot Project in 2014. Several studies have shown that incorporating geriatric‐specific variables into clinical databases is crucial for assessing surgical quality in elderly patients [7, 8, 9]. Similarly, the JSGS conducted a geriatric pilot study in 2018 [10], collecting clinical data from over 5000 surgical cases, including geriatric‐specific variables, across 22 academic and community hospitals. This study developed five risk models for geriatric outcomes: (1) postoperative delirium, (2) physical function on postoperative day 30, (3) fall risk at discharge, (4) discharge to a location other than home with social services, and (5) functional decline at discharge. These models were implemented as risk calculators on the NCD web system (http://www.ncd.or.jp). However, the pilot study could not evaluate the relationship between geriatric factors and surgical mortality or morbidity due to the low incidence of these events. To overcome this limitation, 22 geriatric‐specific variables were added to the NCD registry in 2021 (details of these variables are provided in Table S1).

This study used the large‐sale NCD geriatric dataset to develop and validate prediction models for 30‐day mortality and major complications (Clavien–Dindo classification 3b or above). It also examined the influence of geriatric‐specific factors on surgical outcomes through statistical modeling. Additionally, this study is the first to explore the incidence of geriatric‐specific outcomes, such as changes in social environment, functional status, and psychosocial well‐being, across seven major gastroenterological surgeries. The findings are essential for providing patient‐centered care for elderly patients and ensuring the quality of surgical care.

Overall, this study highlights the importance of creating a comprehensive database that includes geriatric‐specific risk factors and outcomes.

## 2 Methods

The study protocol was approved by the Ethics Committee of Fukushima Medical University in accordance with the Declaration of Helsinki (Approval No. Ippan‐2020‐033).

### 2.1 Data Source and Patient Selection

**FIGURE 1:**
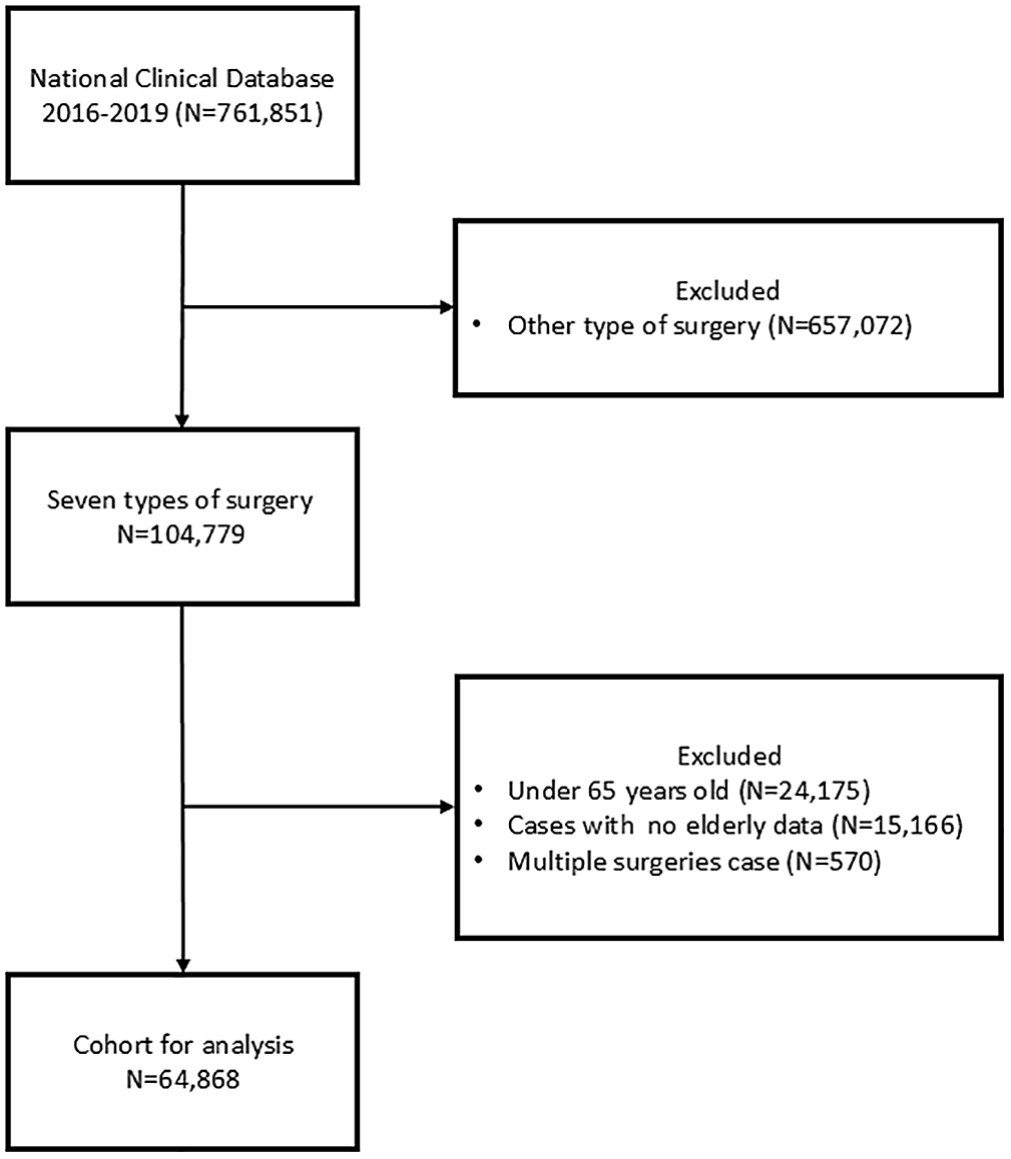
Patient flow.

Perioperative data for this study were obtained from the NCD for patients who underwent seven major gastroenterological surgeries in 2021: low anterior resection (LAR), pancreaticoduodenectomy (PD), distal gastrectomy (DG), esophagectomy (ESO), hepatectomy (HEP), right hemicolectomy (RC), and total gastrectomy (TG). Clinical data, including at least one entry for geriatric variables and outcomes, were extracted for individuals aged 65 years and older (Figure 1).

### 2.2 Preoperative Geriatric‐Specific Variables (Preoperative Risk Factors and Postoperative Outcome Registry in NCD)

The NCD Geriatric Surgery Pilot Project began collecting 22 geriatric‐specific variables for patients aged 65 and older in 2019 among 21 hospitals volunteering to join the project. The NCD Geriatric study based on the project [10] identified 11 of these variables were relevant in the predictive modeling of geriatric outcomes. Consequently, these variables were selected as data items to be widely collected in from all hospitals participating in the NCD. In addition, preoperative depression status, which is increasingly recognized as an important factor in the care of elderly patients [11], was added to the NCD geriatric data collection. In total, 12 geriatric variables are now being collected in the NCD JSGS registry since 2021 (Table S1). Seven preoperative variables are used for comprehensive geriatric assessment: “Origin status from home” and “Preoperative ADL (independent/dependent)” for functional status, “History of dementia” for cognitive status, “Surrogate consent” for decision‐making ability, “Fall history” and “Use of mobility aid” for mobility status, and “Depression” for mental health. These preoperative geriatric‐variables were recorded by the attending surgeon or preoperative nurse based on patient (or family) self‐report and/or a referral letter from the previous physician. Surrogate consent is recorded when a proxy provides consent instead of the patient, regardless of the underlying reason (e.g., cognitive dysfunction, delirium, communication difficulty, or emergency operation). For geriatric‐specific outcomes, six variables were collected in the NCD registry: “Physical function comparing preoperative baseline to 30 days postoperatively”, “Functional status on discharge”, “Fall risk on discharge”, “Postoperative new use of mobility aid”, “Postoperative delirium,” and “hospital destination with or without the need for additional services”. Postoperative delirium is defined as “clinically diagnosed delirium within 30 days postoperatively,” as recorded by the attending physician or nurse based on chart documentation. No standardized assessment tools (e.g., CAM‐ICU, 4AT) were uniformly used.

### 2.3 Outcome Measures and Creating Risk Models

The surgical outcomes for modeling were 30‐day mortality and major postoperative complications, defined as Clavien–Dindo grade 3b or above [12]. Risk models for these two outcomes were developed using the 2021 NCD dataset, which included geriatric variables. In addition, the six geriatric‐specific outcomes were evaluated for the seven gastroenterological surgeries.

### 2.4 Statistical Analysis

Descriptive statistics were used to summarize the demographic, clinical, and laboratory characteristics of the study participants. Categorical variables were presented as numbers and percentages. A random sample of 70% of the cohort was selected as the development cohort, with the remaining 30% designated as the validation cohort. Multivariable logistic regression with backward stepwise selection was performed to develop risk models in the development cohort based on potential predictors. Cases with missing values were excluded from the analysis. Age categories (65–74, 75–84, > 85 years) were treated as continuous variables. Other variables included in the analysis were those preselected by the investigators as clinically relevant. The discrimination and calibration of the developed models were assessed by applying them to the testing cohort and predicting the outcomes for each patient. C‐statistic values (areas under the receiver operating characteristic [ROC] curve) were reported, and calibration plots along with the Hosmer–Lemeshow (HL) test were used to assess goodness‐of‐fit. All tests were two‐sided, with *p* < 0.05 considered statistically significant. Analyses were performed using SAS version 9.4 (SAS Institute, Cary, NC, USA).

## 3 Results

### 3.1 Patient Characteristics, Preoperative Risk Profiles, and Laboratory Data of the Study Population

In 2021, the NCD recorded 761 851 gastroenterological surgery cases across 2367 facilities. From this, 64 868 cases were selected, each containing at least one geriatric variable for patients aged 65 and older (Figure S1). These cases came from 1853 facilities, representing 78% of the NCD's gastroenterological surgery facilities. The study cohort characteristics are summarized in Table 1. The majority of patients in this study cohort were in their 70s (52.7%), with those over 80 accounting for 29.6% of the total. In terms of surgical procedures, the cohort had a higher proportion of PDs (11.5%; 7439 cases) and RHCs (22.6%; 14 691 cases) compared to the overall NCD registry, which includes all age groups [2].

**TABLE 1:** Profiles and laboratory data of the entire study population and outcome groups.

Basic demography	Entire study population		Development cohort		Validation cohort	
	*n* = 64 868		*n* = 45 409		*n* = 19 459	
	No. of patients (%)		No. of patients (%)		No. of patients (%)	
General variables						
Age						
65–69	11 495	17.7%	8058	17.7%	3437	17.7%
70–74	18 704	28.8%	13 155	29.0%	5549	28.5%
75–79	15 489	23.9%	10 786	23.8%	4703	24.2%
80–84	11 775	18.2%	8187	18.0%	3588	18.4%
85–89	5752	8.9%	4055	8.9%	1697	8.7%
90	1653	2.5%	1168	2.6%	485	2.5%
Sex						
Female	23 845	36.8%	16 676	36.7%	7169	36.8%
Body mass index						
< 18.5	8875	13.7%	6211	13.7%	2664	13.7%
18.5‐, < 25	42 844	66.0%	30 066	66.2%	12 778	65.7%
25‐, < 30	11 515	17.8%	7999	17.6%	3516	18.1%
30—	1608	2.5%	1119	2.5%	489	2.5%
Missing	1026	1.6%	14	0.0%	12	0.1%
ASA PS classification						
1 and 2	52 219	80.5%	36 489	80.4%	15 730	80.8%
3, 4, and 5	12 649	19.5%	8920	19.6%	3729	19.2%
Preoperative ADL						
Independent	59 980	92.5%	41 964	92.4%	18 016	92.6%
Emergent surgery	1771	2.7%	1247	2.7%	524	2.7%
Geriatric variables						
Origin status from home						
From home	54 544	84.1%	38 133	84.0%	16 411	84.3%
Not from home	4548	7.0%	3232	7.1%	1316	6.8%
Missing	5776	8.9%	4044	8.9%	1732	8.9%
Fall history						
Yes	2034	3.1%	1433	3.2%	601	3.1%
No	57 494	88.6%	40 235	88.6%	17 259	88.7%
Missing	5340	8.2%	3741	8.2%	1599	8.2%
History of Dementia						
Yes	3270	5.0%	2347	5.2%	923	4.7%
No	56 573	87.2%	39 532	87.1%	17 041	87.6%
Missing	5025	7.7%	3530	7.8%	1495	7.7%
Depression						
Yes	758	1.2%	538	1.2%	220	1.1%
No	58 843	90.7%	41 177	90.7%	17 666	90.8%
Missing	5267	8.1%	1573	3.5%	3694	19.0%
Surrogate consent						
No; the patients signed his/her own consent	54 344	83.8%	38 047	83.8%	16 297	83.8%
Yes; consent is signed by a surrogate	5386	8.3%	3769	8.3%	1617	8.3%
Missing	5138	7.9%	3593	7.9%	1545	7.9%
Use of mobility aid						
Yes	7629	11.8%	5369	11.8%	2260	11.6%
No	54 949	84.7%	38 456	84.7%	16 493	84.8%
Missing	2290	3.5%	1584	3.5%	706	3.6%
Preoperative comorbidity variables						
Dyspnea	1160	1.8%	793	1.7%	367	1.9%
Respirator	222	0.3%	152	0.3%	70	0.4%
COPD	2976	4.6%	2064	4.5%	912	4.7%
Hypertension	33 793	52.1%	23 646	52.1%	10 147	52.1%
Diabetes mellitus	16 100	24.8%	11 290	24.9%	4810	24.7%
Congestive heart failure	634	1.0%	449	1.0%	185	1.0%
PVD with a symptom	265	0.4%	176	0.4%	89	0.5%
Acute renal failure	98	0.2%	71	0.2%	27	0.1%
Hemodialysis	553	0.9%	402	0.9%	151	0.8%
Cerebrovascular disease	4151	6.4%	2928	6.4%	1223	6.3%
Steroid use	906	1.4%	617	1.4%	289	1.5%
Weight loss	2981	4.6%	2116	4.7%	865	4.4%
Disseminated cancer	1301	2.0%	939	2.1%	362	1.9%
Laboratory values						
Hemoglobin < 13.5 mg/dL for males, < 12.5 mg/dl for females	36 609	56.4%	25 688	56.6%	10 921	56.1%
Platelet < 15 × 10^4^/ul	4926	7.6%	3414	7.5%	1512	7.8%
Albumin < 3.5 g/dL	17 024	26.2%	11 938	26.3%	5086	26.1%
AST > 35 IU/L	6892	10.6%	4812	10.6%	2080	10.7%
Serum Na < 137 mEq/L	5656	8.7%	3981	8.8%	1675	8.6%
BUN > 25 mg/dL	4926	7.6%	3440	7.6%	1486	7.6%
PT INR > 1.25	2029	3.1%	1449	3.2%	580	3.0%
Procedure						
Distal gastrectomy	18 483	28.5%	12 902	28.4%	5581	28.7%
Low anterior resection	10 602	16.3%	7437	16.4%	3165	16.3%
Right hemicolectomy	14 691	22.6%	10 383	22.9%	4308	22.1%
Total gastrectomy	6528	10.1%	4594	10.1%	1934	9.9%
Pancreaticoduodenectomy	7439	11.5%	5102	11.2%	2337	12.0%
Esophagectomy	3279	5.1%	2302	5.1%	977	5.0%
Hepatectomy	3846	5.9%	2689	5.9%	1157	5.9%

Abbreviations: ADL, activity of daily living; ASA, American Society of Anesthesia; AST, aspartate aminotransferase; BUN, Blood urea nitrogen; COPD, chronic obstructive pulmonary disease; INR, international normalized ratio; PS, Performance Status; PT, prothrombin time; PVD, peripheral arterial disease.

Regarding geriatric risk factors, 84.1% of patients were admitted to the hospital from home, and 92.5% were functionally independent in activities of daily living (ADL). There were 2034 cases (3.1%) with a history of falls, 7629 cases (11.8%) using mobility aids, 3270 cases (5.0%) with dementia, and 758 cases (1.2%) with depression. Detailed information on these factors for each surgical procedure is provided in Table S2.

### 3.2 Mortality, Morbidity, and Geriatric‐Specific Outcomes in Seven Major Gastroenterological Surgeries

The 30‐day mortality rate and major complication rate for all procedures were 1.8% and 11.3%, respectively. The incidence of these outcomes and geriatric‐specific outcomes for each surgical procedure is summarized in Table 2. Notably, both PD and ESO had significantly higher rates of major complications (PD 23.7%, ESO 23.6%), while RHC and TG had higher postoperative mortality rates (RHC 2.4%, TG 2.6%) compared to other procedures. The 30‐day mortality rates for these seven major surgeries in elderly patients were higher than those in the NCD registry cohort, which includes all age groups [2]. For the six geriatric‐specific outcomes, physical functional decline on postoperative day 30 was more common in ESO (28.6%) and PD (19.8%). Regarding ADL at discharge, 11.9% of patients across all surgical procedures were either partially independent or totally dependent, with the highest rate observed in RHC (17.5%). The fall risk at discharge was classified as high in 16% of patients across all procedures. New use of a mobility aid at discharge, discharge to a destination other than home, and postoperative delirium occurred in 4.2%, 7.1%, and 19.5% of patients, respectively, across all surgical procedures. Among discharge destinations, ESO (9.0%) had the highest rate of patients being transferred to another hospital or clinic. Patients who underwent RHC showed a higher risk of falls (20.0%), new use of mobility aids (4.9%), and being transferred to a long‐term care facility (1.4%) compared to other procedures.

**TABLE 2:** Incidence of surgical and geriatric outcomes according to surgical procedure.

Surgical outcomes and geriatric outcomes	Surgical procedure															
	LAR (*n* = 10 602)	%	PD (*n* = 7439)	%	DG (*n* = 18 483)	%	ESO (*n* = 3279)	%	HEP (*n* = 3846)	%	RHC (*n* = 14 691)	%	TG (*n* = 6528)	%	Total (*n* = 64 868)	%
Postoperative complication of Grade 3b or above on POD30	1065	10.0	1765	23.7	1393	7.5	773	23.6	566	14.7	996	6.8	775	11.9	7333	11.3
Postoperative mortality on POD30	103	1.0	146	2.0	229	1.2	63	1.9	97	2.5	353	2.4	172	2.6	1163	1.8
Physical function comparing the preoperative baseline to 30 days postoperatively																
Death at discharge	63	0.6	82	1.2	127	0.7	29	0.9	61	1.6	242	1.7	89	1.4	693	1.1
Improved physical function	848	8.3	325	4.6	1394	7.8	125	4.0	201	5.4	1442	10.2	439	7.0	4774	7.7
Diminished physical function	1190	11.7	1410	19.8	2270	12.8	905	28.6	474	12.7	1808	12.8	1075	17.1	9132	14.6
Similar physical function	7864	77.3	5199	73.0	13 643	76.7	2046	64.7	2902	78.1	10 417	73.6	4536	72.3	46 607	74.7
Unknown details	211	2.1	109	1.5	349	2.0	55	1.7	80	2.2	246	1.7	139	2.2	1189	1.9
Functional status on discharge																
Independent	9168	88.8	6392	88.7	15 540	86.5	2807	88.3	3353	89.5	11 393	79.7	5453	85.9	54 106	85.8
Partial dependent	890	8.6	574	8.0	1790	10.0	257	8.1	243	6.5	2048	14.3	589	9.3	6391	10.1
Total dependent	123	1.2	57	0.8	308	1.7	28	0.9	25	0.7	451	3.2	110	1.7	1102	1.7
Death at discharge	88	0.9	137	1.9	208	1.2	58	1.8	94	2.5	331	2.3	161	2.5	1077	1.7
Unknown details	61	0.6	47	0.7	111	0.6	29	0.9	33	0.9	79	0.6	32	0.5	392	0.6
Fall risk on discharge																
High risk	1381	13.5	1063	14.9	2807	15.7	503	15.9	477	12.9	2828	20.0	968	15.4	10 027	16.1
Low risk	6302	61.6	4422	62.1	10 665	59.8	2011	63.4	2304	62.2	7905	55.9	3660	58.3	37 269	59.7
No fall risk evaluation performed	997	9.7	678	9.5	1816	10.2	277	8.7	407	11.0	1426	10.1	676	10.8	6277	10.0
Not applicable	1547	15.1	957	13.4	2541	14.3	379	12.0	518	14.0	1982	14.0	976	15.5	8900	14.2
Postoperative new use of mobility aid																
No	9897	96.2	6862	95.7	17 132	96.1	3057	96.4	3583	96.2	13 513	95.1	6002	95.3	60 046	95.8
Yes	390	3.8	305	4.3	696	3.9	113	3.6	143	3.8	697	4.9	296	4.7	2640	4.2
Discharge destination																
Discharge to home	9594	92.4	6627	91.6	16 500	91.4	2800	87.6	3468	92.2	12 427	86.6	5719	89.9	57 135	90.2
Transferring to a different hospital or clinic	394	3.8	354	4.9	821	4.5	287	9.0	151	4.0	861	6.0	317	5.0	3185	5.0
Transferring to a long‐term care health facility	221	2.1	43	0.6	352	1.9	12	0.4	23	0.6	530	3.7	103	1.6	1284	2.0
Transferring to another ward in the same hospital	71	0.7	56	0.8	122	0.7	22	0.7	20	0.5	167	1.2	51	0.8	509	0.8
Death at discharge	91	0.9	139	1.9	217	1.2	60	1.9	94	2.5	332	2.3	162	2.5	1095	1.7
Unknown details	17	0.2	16	0.2	42	0.2	14	0.4	7	0.2	41	0.3	10	0.2	147	0.2
Postoperative delirium																
No	8474	81.7	6047	82.7	14 619	80.9	2640	81.9	3089	81.8	11 347	78.9	5159	80.4	51 375	80.8
Yes	1899	18.4	1261	18.6	3451	19.2	585	18.4	685	18.9	3040	21.1	1257	19.8	12 178	19.5

*Note:* The number of cases classified as “death at discharge” differs across the listed outcome categories. These discrepancies may result from differences in the timing of outcome measurement (in‐hospital death vs. 30‐day postoperative death) and may also be partially influenced by incomplete registry documentation or missing data.

Abbreviations: LAR, low anterior resection; PD, pancreaticoduodenectomy; DG, distal gastrectomy; ESO, esophagectomy; HEP, hepatectomy; RHC, right‐hemi colectomy; and TG, total gastrectomy.

### 3.3 Importance of Geriatric‐Specific Risk Factors in Predicting 30‐Day Mortality or Major Postoperative Complications

**FIGURE 2:**
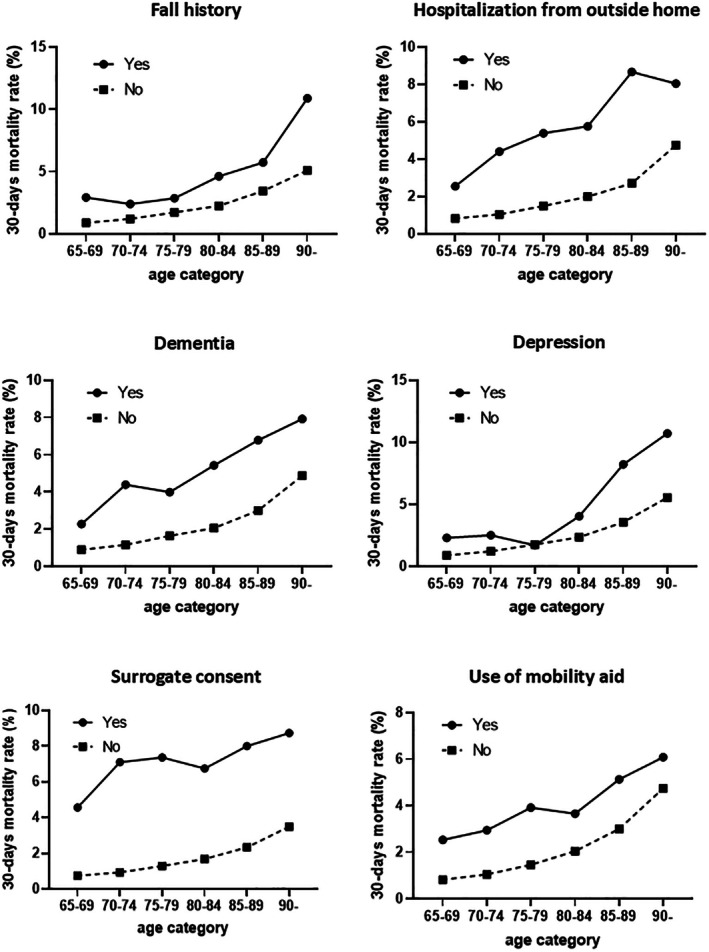
Impact of geriatric factors on 30‐day mortality. The 30‐day mortality rates for patients with and without a geriatric factor are shown by age category.

The impact of geriatric factors on the prediction model for 30‐day mortality or major complications is shown in Tables 3, S3 and Figure 2. Figure 2 highlights that all six factors negatively impacted postoperative mortality across all age groups. Notably, a significant difference in mortality rates was observed between those with or without “Surrogate consent” and “Hospitalization from outside the home.”

**TABLE 3:** Risk models of mortality and postoperative complications.

		The 30‐day mortality				Major complication (Clavien–Dindo 3b or above)			
Term		B. coefficient	*p*	OR	95% CI	B. coefficient	*p*	OR	95% CI
Age	65–69	Reference	—	—	—				
	70–74	0.181	0.2513	1.20	0.88–1.63				
	75–79	0.481	0.0019	1.62	1.20–2.19				
	80–84	0.744	< 0.0001	2.10	1.55–2.86				
	85–89	0.957	< 0.0001	2.60	1.86–3.64				
	90—	1.329	< 0.0001	3.78	2.53–5.63				
Sex	Male	Reference	—	—	—	Reference	—	—	—
	Female	−0.4948	< 0.0001	0.61	0.51–0.73	−0.470	< 0.001	0.625	0.56–0.70
ASA PS classification: 1 and 2	1 and 2	Reference		—	—	Reference	—	—	—
3, 4, and 5	3, 4, and 5	0.515	< 0.001	1.67	1.41–1.99	0.370	< 0.001	1.447	1.29–1.62
Preoperative ADL	Independent	Reference		—	—	Reference	—	—	—
	Dependent	0.656	< 0.001	1.93	1.56–2.38	0.328	< 0.001	1.389	1.18–1.63
Dyspnea	No symptom	Reference		—	—				
	Resting dyspnea	0.975	0.0014	2.65	1.46–4.83				
	Effort dyspnea	0.546	0.0069	1.73	1.16–2.57				
Ventilator dependent within 48 h	No	Reference		—	—	Reference	—	—	—
	Yes	1.166	< 0.0001	3.21	1.86–5.54	0.979	< 0.001	2.663	1.70–4.17
COPD	No					Reference	—	—	—
	Yes					0.346	< 0.001	1.409	1.17–1.70
Congestive heart failure within 30 days	No	Reference		—	—				
	Yes	0.587	0.004	1.80	1.21–2.68				
Hypertension with no treatment	No					Reference	—	—	—
	Yes					0.013	0.9347	1.013	0.74–1.39
Hypertension with medication	No					Reference	—	—	—
	Yes					0.170	0.0007	1.185	1.07–1.31
Previous PVD with symptom	No	Reference		—	—				
	Yes	0.626	0.0832	1.87	0.92–3.80				
Hemodialysis within 14 days	no	Reference		—	—	Reference	—	—	—
	Yes	1.266	< 0.0001	3.55	2.40–5.25	0.717	< 0.001	2.048	1.48–2.82
Cerebrovascular disease	No	Reference		—	—				
	COMA lasting more than 24 h	1.362	0.162	3.90	0.58–26.3				
	CVA lasting more than 72 h	−0.462	0.1401	0.63	0.34–1.16				
	RIND	0.795	0.0411	2.214	1.03–4.75				
	TIA	0.583	0.1045	1.792	0.89–3.62				
	Unknown details	0.010	0.9509	1.01	0.74–1.38				
Depression	No	Reference		—	—				
	Yes	0.517	0.0401	1.68	1.02–2.75				
Surrogate consent	No	Reference		—	—	Reference	—	—	—
	Yes	0.647	< 0.0001	1.91	1.55–2.35	0.415	< 0.001	1.52	1.30–1.76
Origin status	Hospitalization from home	Reference		—	—	Reference	—	—	—
	Hospitalization from outside the home	0.217	0.0447	1.24	1.01–1.54	0.243	0.0022	1.28	1.09–1.49
Emergent surgery	No	Reference		—	—	Reference	—	—	—
	Yes	1.419	< 0.0001	4.13	3.22–5.31	1.114	< 0.001	3.047	2.50–3.71
Advanced cancer with dissemination	No	Reference		—	—	Reference	—	—	—
	Yes	1.236	< 0.0001	3.44	2.52–4.71	0.499	0.0002	1.647	1.27–2.14
Weight loss	No	Reference		—	—				
	Yes	0.567	< 0.0001	1.76	1.36–2.28				
Platelet < 15 × 10^4^/ul	No	Reference		—	—	Reference	—	—	—
	Yes	0.408	0.0003	1.50	1.20–1.88	0.241	0.0024	1.272	1.09–1.49
Albumin < 3.5 g/dl	No	Reference		—	—	Reference	—	—	—
	Yes	0.584	< 0.0001	1.79	1.51–2.14	0.341	< 0.001	1.407	1.26–1.57
AST > 35 IU/L	No	Reference		—	—	Reference	—	—	—
	Yes	0.464	< 0.0001	1.59	1.31–1.94	0.205	0.0041	1.228	1.07–1.41
BUN > 25 mg/dL	No	Reference		—	—	Reference	—	—	—
	Yes	0.385	0.0003	1.47	1.19–1.81	0.301	< 0.001	1.351	1.16–1.57
PT INR > 1.25	No	Reference		—	—	Reference	—	—	—
	Yes	0.571	< 0.0001	1.77	1.37–2.30	0.458	< 0.001	1.581	1.30–1.92
Type of surgery	Distal gastrectomy	Reference		—	—	Reference	—	—	—
	Low anterior resection	−0.027	0.8693	0.97	0.71–1.34	0.834	< 0.001	2.302	1.98–2.67
	Pancreaticoduodenectomy	1.030	< 0.0001	2.80	2.12–3.71	0.744	< 0.001	2.105	1.77–2.50
	Esophagectomy	1.050	< 0.0001	2.86	1.96–4.15	1.277	< 0.001	3.585	2.97–4.32
	Hepatectomy	1.314	< 0.0001	3.72	2.72–5.09	0.407	0.0006	1.502	1.19–1.89
	Right hemicolectomy	−0.079	0.5201	0.92	0.73–1.18	−0.076	0.36	0.927	0.79–1.09
	Total gastrectomy	0.839	< 0.0001	2.31	1.77–3.02	0.511	< 0.001	1.666	1.39–1.99
Intercept		−5.92	< 0.001	—	—	−3.92	< 0.001	—	—
H‐L score		0.198				0.000277			
C‐index		0.84 (0.82–0.86)				0.68 (0.66–0.70)			

Abbreviations: ADL, activity of daily living; ASA, American Society of Anesthesia; AST, aspartate aminotransferase; BUN, Blood urea nitrogen; CI, confidence interval; COPD, chronic obstructive pulmonary disease; INR, International normalized ratio; OR, odds ratio; PS, Performance Status; PT, prothrombin time.

Risk models for 30‐day mortality and major complications were developed using multivariate logistic analysis (Table 3). The model for 30‐day mortality included 22 predictors, with three geriatric‐specific variables identified as significant: surrogate consent (odds ratio [OR] 1.91; 95% confidence interval [CI] 1.55–2.35), depression (OR 1.68; 95% CI 1.02–2.75), and hospitalization from outside the home (OR 1.24; 95% CI 1.01–1.54). The model for major complications retained 16 predictors, with two geriatric‐specific variables as significant predictors: surrogate consent (OR 1.52; 95% CI 1.30–1.76) and hospitalization from outside the home (OR 1.28; 95% CI 1.09–1.49).

**FIGURE 3:**
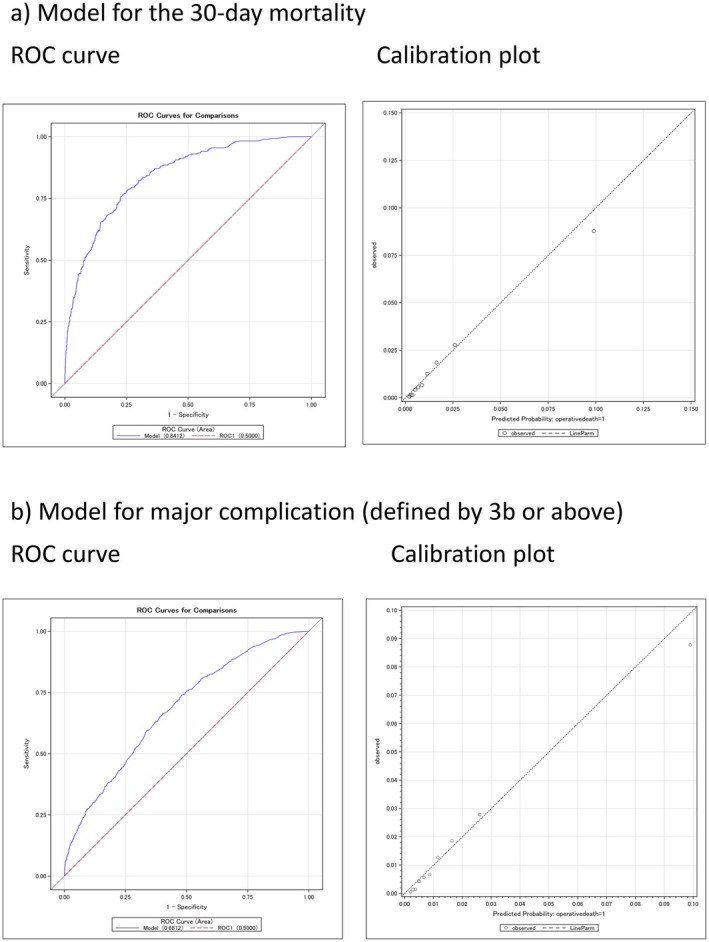
ROC curve and calibration plots of the developed risk models.

The C‐index, representing the area under the ROC curve, and the 95% CI were calculated using the validation cohort. The C‐indices of 30‐day mortality and major complications were 0.84 (95% CI 0.82–0.86) and 0.68 (95% CI 0.66–0.70), respectively (Figure 3), which were similar to those of established risk models developed using NCD registry data without geriatric‐specific variables [13, 14, 15, 16, 17, 18, 19, 20].

Calibration plots for the risk model predicting each outcome are shown in Figure 3. The predicted surgical outcomes closely matched the actual probabilities across both low‐ and high‐risk groups, demonstrating consistent calibration in both models.

## 4 Discussion

This study developed and validated risk models for 30‐day mortality and major complications in elderly patients undergoing seven major gastroenterological surgeries, using a nationwide surgical registry that covers almost all of Japan. The models showed good predictive performance and reliability. The discrimination performance for predicting 30‐day mortality was high, with a C‐index of 0.84 (95% CI 0.82–0.86), comparable to established models based on general variables rather than geriatric‐specific ones [13, 14, 15, 16, 17, 18, 19, 20]. In the mortality prediction model, three geriatric‐specific factors—surrogate consent (OR 1.91; 95% CI 1.55–2.35), depression (OR 1.68; 95% CI 1.02–2.75), and hospitalization from outside the home (OR 1.24; 95% CI 1.01–1.54)—along with age, were identified as independent predictors. The impact of the three factors on prediction was similar to that of patient age, with those aged 75–79 years having a higher OR (OR 1.62; 95% CI 1.20–2.19) compared to those aged 65–69 years. In addition, this study provides real‐world evidence on geriatric‐specific outcomes, highlighting potential changes in patients' physical functional and social environment following each procedure in Japan.

A key finding of this study is that geriatric‐specific risk factors, such as preoperative depression (mental state), surrogate consent (cognitive status), and hospitalization from outside the home (origin status), negatively impacted 30‐day mortality in major gastroenterological surgeries. While other geriatric risk factors, including fall history, dementia, and use of mobility aids, were not identified as independent prognosticators, a correlation with postoperative mortality or complications was observed in univariate analysis (Table S3). The NCD Geriatric Surgery Pilot Project [10] previously indicated a close relationship between geriatric‐specific factors and outcomes, but due to its small sample size, the association with 30‐day mortality or major complications was not fully explored. This study helps address the limitations of the initial NCD Geriatric Surgery Pilot Project.

Patient age is widely recognized as a strong predictor of postoperative mortality and is included in the established NCD risk calculators for all seven major gastroenterological surgeries. In this study, as expected, age was identified as an independent predictor of 30‐day mortality but not of major postoperative complications. When comparing the contribution of age and geriatric‐specific risk factors to the prediction using ORs in the developed risk model, the ORs for the three geriatric‐specific risk factors were similar to that of age for patients aged 75–79 years (Table 3). In addition, the 30‐day mortality rate was higher for those with a geriatric risk factor across all age groups, as shown in Figure 2, indicating that geriatric‐specific factors add to the risk stratification for surgical outcomes in elderly patients.

Studies have demonstrated that frailty associated with aging is an independent risk factor for surgical outcomes [21, 22]. However, there is limited evidence on which geriatric‐specific factors are significant for postoperative mortality or morbidity. Brian et al. investigated the impact of geriatric‐specific risk factors on adjusting risk for 30‐day mortality and serious morbidity or mortality in general–vascular and orthopedic surgery, finding that surrogate consent, fall history, and preoperative use of mobility aids were key factors [9]. The value of this study is in identifying which geriatric factors are most important for predicting mortality or morbidity in gastroenterological surgeries. Among the three risk factors, surrogate consent and hospitalization from outside the home were also found to be independent predictors for major complications (Table 3). As shown in Figure 2, the difference in mortality rates between patients with and without “Surrogate consent” or “Hospitalization form outside the home” appeared more pronounced than other factors. Surrogate consent may indicate significant cognitive decline beyond a history of dementia and could be linked to declines in physical function, making it a potentially important indicator of vulnerability in elderly patients. The discrepancy between the prevalence of surrogate consent (8.3%) and dementia (5.0%), as shown in Table 1, suggests that surrogate consent may capture not only severe cognitive impairment but also other conditions, such as delirium, emergency surgery, or cultural factors in Japan where family involvement in medical decision‐making is common. Regarding preoperative depression, which was newly added to the NCD registry's geriatric dataset, this study is the first to show that it is an independent predictor of postoperative surgical outcomes. It is well‐known that postoperative depression is a common cause of morbidity or mortality following surgery [23]. Preoperative depression is recognized as a risk factor for postoperative depression [11], which can negatively affect healthcare outcomes after surgery. The results emphasize the importance of assessing preoperative depressive status and support its inclusion in the NCD registry. These findings highlight that the addition of geriatric‐specific variables to the NCD registry is crucial for a thorough geriatric assessment.

In this study, the prevalence of preoperative geriatric factors was lower than that reported in community‐based studies. For example, the prevalence of depression in our cohort was 1.2%, whereas community‐based studies of older adults using standardized assessment tools such as the Geriatric Depression Scale have reported rates of 23.4%–34.2% [24]. Because the NCD data collection relies on medical record abstraction or attending surgeon assessment rather than standardized questionnaires, underreporting is possible. Similarly, the prevalence of fall history in our cohort (3.1%) was markedly lower than that reported in a community‐based study (18.3%) [25] as well as a prior surgical geriatric cohort (20.0%) [9]. Taken together, the lower prevalence of geriatric factors in our cohort likely reflects both selection bias toward healthier surgical candidates and limited ascertainment sensitivity. This study is the first to provide real‐world data on geriatric outcomes for each of the seven major gastroenterological surgeries in Japan (Table 2). For physical function changes after surgery, ESO (28.64%) and PD (19.79%) showed higher rates of diminished physical function compared to the other procedures. ESO also had the highest rate of patients transferred to a different hospital or clinic (8.98%). Unexpectedly, postoperative outcomes for RHC were worse than for other procedures, with higher mortality (2.4%) and fall risk (20.0%). It is important to consider that RHC patients were more frail, with higher rates of total dependence before surgery or surrogate consent (Table S2). This evidence underscores the potential changes in physical function or the need for social support after surgery, which is important for patient‐centered care, particularly in shared decision‐making before surgery [26, 27]. Since elderly patients are at a higher risk of functional decline or loss of independence postoperatively, healthcare providers must focus on the outcomes most important to these patients. Some elderly individuals may prioritize overall well‐being and quality of life over the length of life after surgery. Traditionally, surgical quality has been evaluated based on prognosis and perioperative morbidity and mortality [13, 14, 15, 16, 17, 18, 19, 20]. To ensure high‐quality surgery for elderly patients, it is essential to evaluate outcomes using geriatric metrics, such as postoperative functional status (ADL, need for new mobility aids), psychosocial well‐being (postoperative depression), and social environment (discharge destination). During shared decision‐making, the medical team must provide sufficient information to support collaborative decisions, including not only the potential benefits of surgery, complications, and mortality but also the risks of functional decline and the need for life support.

This study has several limitations. First, while this study conducted internal validation of the newly developed risk models, external validation using an independent dataset is crucial. Validation with a database outside Japan is also essential to evaluate the model's generalizability. Second, restricting inclusion to patients with at least one geriatric variable recorded may have introduced information bias. Specifically, cases with more comprehensive recording might reflect institutions with more attention to frailty or complexity, which could influence outcome estimates. Regarding missing data, Table S4 summarizing the proportion of missing data for each variable showed missingness was not concentrated in specific surgical or demographic groups. Third, while it examined geriatric‐specific outcomes at discharge, it is likely that these outcomes are related to long‐term results after discharge. Brian et al. identified a correlation between loss of independence at discharge and increased risks of readmission and mortality following surgical procedures in older patients [28]. Further research is needed to assess long‐term outcomes after discharge in elderly patients who have undergone surgery in Japan.

## 5 Conclusion

Risk models for 30‐day mortality and major complications in elderly patients undergoing gastroenterological surgery were developed and validated, demonstrating strong predictive performance and reliability. In the mortality model, three geriatric‐specific risk factors—“Hospitalization from outside the home”, “Surrogate consent,” and “Depression”—along with age, were identified as significant contributors. Incorporating geriatric‐specific factors along with age identified clinically relevant predictors of mortality and morbidity, reinforcing the importance of geriatric assessment in elderly surgical patients. Overall, this study highlights the importance of creating a comprehensive database that incorporates geriatric‐specific risk factors and outcomes.

## Author Contributions

**Naoya Sato:** investigation, validation, writing – original draft, writing – review and editing, methodology, data curation. **Hiraku Kumamaru:** conceptualization, methodology, software, data curation, validation, formal analysis, supervision, writing – review and editing. **Mitsukazu Gotoh:** conceptualization, methodology, data curation, funding acquisition, writing – review and editing, validation. **Yoshihiro Kakeji:** conceptualization, investigation, writing – review and editing, resources, validation, funding acquisition. **Yuko Kitagawa:** conceptualization, validation, investigation, writing – review and editing, resources, funding acquisition. **Yasuyuki Seto:** conceptualization, investigation, funding acquisition, validation, resources, writing – review and editing. **Hiromi Rakugi:** writing – review and editing, conceptualization, investigation, funding acquisition, validation, resources. **Masahiro Akishita:** writing – review and editing, validation, formal analysis, investigation, conceptualization. **Kazue Nakajima:** investigation, conceptualization, writing – review and editing, data curation, validation. **Arata Takahashi:** methodology, software, data curation, validation, visualization, formal analysis. **Hiroaki Miyata:** methodology, software, data curation, formal analysis, validation, supervision. **Shigeru Marubashi:** supervision, project administration, conceptualization, investigation, visualization, funding acquisition, writing – review and editing, methodology, data curation, formal analysis, validation.

## Funding

This work was funded by the Health and Labor Sciences Research Grants (21EA0401).

## Ethics Statement

The study protocol was approved by the Japanese Society of Gastroenterological Surgery and the Ethics Committee of Kobe University (approval number 20190128).

## Conflicts of Interest

Yuko Kitagawa is Editor in Chief of Annals of Gastroenterological Surgery. Yoshihiko Kakeji is an editorial board member of the Annals of Gastroenterological Surgery. Hiraku Kumamaru, Arata Takahashi, and Hiroaki Miyata are affiliated with the Department of Healthcare Quality Assessment at the University of Tokyo. The department is a social collaboration department supported by National Clinical Database, Johnson & Johnson K.K., Nipro Corporation, and Intuitive Surgical Sàrl.

## Supporting information


**Table S1:** Geriatric surgery variables and definitions.

**Table S2:** Detailed information on preoperative geriatric factors by types of surgical procedure.

**Table S3:** Relationship between geriatric factors and postoperative outcomes.

**Table S4:** Number of missing data by age and surgical procedure.

## Data Availability

Research data are not shared. We used data from the National Clinical Database (NCD) registry in Japan. The NCD committee prohibits us from sharing the data with anyone outside this research group.
