# Supplementary material for: Nationwide Real‐World Modeling of Surgical Outcomes in Elderly Patients: Incorporating Geriatric‐Specific Risk Factors Into Prediction of Mortality and Morbidity
Source: Ann Gastroenterol Surg. 2026 Jan 11;10(3):904–19. doi: 10.1002/ags3.70164 (PMC13178288; doi:10.1002/ags3.70164)
Supplement: Supplementary file 1 — Table S1: Geriatric surgery variables and definitions. [file AGS3-10-904-s001.docx]

| Supplementary table_1. Geriatric surgery variables and definitions | |
| --- | --- |
|  | variable intent/definitions |
| Preoperative variables |  |
| Hospitalization from home | To differentiate patients who are admitted from home from those not from home (facility or other) |
| Fall history | To capture patients who experienced a fall within 1 year |
| History of Dementia | To identify a diagnosis of cognitive impairment or history of dementia, including dementia, Alzheimer's disease, vascular dementia, Parkinson's disease, and so on. |
| Surrogate consent | to identify patients with a surrogate consent reflecting competency status, i.e. a patient with severe cognitive impairment that renders him or her incapable of understanding the informed consent discussion. |
| Use of mobility aid | To quantify baseline mobility based on use of a walking aid, such as a walker, cane, or requires a wheel chair or scooter. |
| Depression | To identify patients who have depression on admission |
| Postoperative variables |  |
| Physical function comparing preoperative baseline to 30 days postoperatively | To compare physical function after surgery with preoperative baseline |
| Functional status on discharge | to determine functional status at the time of discharge (ability to perform activities of daily living) |
| Fall risk on discharge | To identify the patients who have the potential risk of fall on discharge due to reduction of physical function |
| Postoperative new use of mobility aid | To define the patients who requires the use of an assistive device on discharge that was not used before or on admission |
| Discharge destination | To identify the patients who need medical care, support or skilled services postoperatively |
| Postoperative delirium | To define the occurrence of postoperative delirium (delirium is present if there are 1 or more episodes of acute confusion during the hospital stay) |
