# Supplementary material for: Nationwide Real‐World Modeling of Surgical Outcomes in Elderly Patients: Incorporating Geriatric‐Specific Risk Factors Into Prediction of Mortality and Morbidity
Source: Ann Gastroenterol Surg. 2026 Jan 11;10(3):904–19. doi: 10.1002/ags3.70164 (PMC13178288; doi:10.1002/ags3.70164)
Supplement: Supplementary file 2 — Table S2: Detailed information on preoperative geriatric factors by types of surgical procedure. [file AGS3-10-904-s004.docx]

| Supplementary table_2. Detailed information on preoperative geriatric factors by types of surgical procedure | | | | | | | | |
| --- | --- | --- | --- | --- | --- | --- | --- | --- |
|  |  | types of surgery procedure | | | | | | |
|  | preoperative geriatric factors | LAR | PD | DG | ESO | HEP | RC | TG |
| 1 | ADL within 30days |  |  |  |  |  |  |  |
|  | independent | 94.2% | 97.3% | 93.2% | 97.9% | 96.6% | 88.1% | 94.6% |
|  | partial dependent | 5.0% | 2.6% | 5.8% | 1.9% | 3.0% | 9.8% | 4.6% |
|  | total dependent | 0.7% | 0.2% | 1.1% | 0.2% | 0.4% | 2.1% | 0.8% |
| 2 | ADL before surgery |  |  |  |  |  |  |  |
|  | independent | 93.7% | 96.9% | 92.5% | 97.8% | 96.4% | 86.5% | 93.8% |
|  | partial dependent | 5.4% | 3.0% | 6.2% | 2.0% | 3.1% | 10.3% | 5.2% |
|  | total dependent | 1.0% | 0.2% | 1.3% | 0.2% | 0.4% | 3.2% | 1.0% |
| 3 | Origin status |  |  |  |  |  |  |  |
|  | Hospitalization from home | 93.4% | 94.4% | 93.3% | 95.5% | 97.0% | 86.8% | 93.7% |
|  | Hospitalization from outside the home | 6.6% | 5.6% | 6.7% | 4.5% | 3.0% | 13.2% | 6.3% |
|  |  |  |  |  |  |  |  |  |
| 4 | Fall history |  |  |  |  |  |  |  |
|  | Yes | 3.0% | 2.7% | 3.5% | 2.3% | 2.3% | 4.7% | 3.2% |
|  | No | 97.0% | 97.3% | 96.5% | 97.7% | 97.7% | 95.3% | 96.8% |
| 5 | History of Dementia |  |  |  |  |  |  |  |
|  | Yes | 4.8% | 2.2% | 5.6% | 1.5% | 2.2% | 9.6% | 4.5% |
|  | No | 95.2% | 97.8% | 94.4% | 98.5% | 97.8% | 90.4% | 95.5% |
| 6 | Depression |  |  |  |  |  |  |  |
|  | Yes | 1.3% | 1.6% | 1.1% | 0.8% | 0.9% | 1.6% | 1.0% |
|  | No | 98.7% | 98.4% | 98.9% | 99.2% | 99.1% | 98.4% | 99.0% |
| 7 | Surrogate consent |  |  |  |  |  |  |  |
|  | Yes | 7.4% | 4.3% | 8.6% | 2.5% | 3.9% | 16.7% | 7.2% |
|  | No | 92.6% | 95.7% | 91.4% | 97.5% | 96.1% | 83.3% | 92.8% |
| 8 | Use of mobility aid |  |  |  |  |  |  |  |
|  | Yes | 10.8% | 7.4% | 12.4% | 5.5% | 8.6% | 18.0% | 11.2% |
|  | No | 89.2% | 92.6% | 87.6% | 94.5% | 91.4% | 82.0% | 88.8% |
| abbreviations; ADL; activity of daily living, LAR; low anterior resection, PD; pancreaticoduodenectomy, DG; distal gastrectomy, ESO; esophagectomy, HEP; hepatectomy, RC; right-hemi colectomy, and TG; total gastrectomy | | | | | | | | |
