# Supplementary material for: Nationwide Real‐World Modeling of Surgical Outcomes in Elderly Patients: Incorporating Geriatric‐Specific Risk Factors Into Prediction of Mortality and Morbidity
Source: Ann Gastroenterol Surg. 2026 Jan 11;10(3):904–19. doi: 10.1002/ags3.70164 (PMC13178288; doi:10.1002/ags3.70164)
Supplement: Supplementary file 3 — Table S3: Relationship between geriatric factors and postoperative outcomes. [file AGS3-10-904-s002.docx]

| Supplementary table_3. Relationship between geriatric factors and postoperative outcomes | | | | | | | | | | |
| --- | --- | --- | --- | --- | --- | --- | --- | --- | --- | --- |
|  |  |  |  | The 30-day mortality | | |  | Major complication (Clavian-Dindo 3b or above) | | |
|  |  |  | N | yes | % | p value |  | yes | % | p value |
| 1 | Hospitalization from home | Yes | 38133 | 581 | 1.52 | <0.001 |  | 1637 | 4.29 | <0.001 |
|  |  | No | 3232 | 189 | 5.85 |  |  | 283 | 8.76 |  |
| 2 | Fall history | Yes | 1433 | 60 | 4.19 | <0.001 |  | 94 | 6.56 | <0.001 |
|  |  | No | 40235 | 722 | 1.79 |  |  | 1839 | 4.57 |  |
| 3 | History of Dementia | Yes | 2347 | 114 | 4.86 | <0.001 |  | 160 | 6.82 | <0.001 |
|  |  | No | 39532 | 668 | 1.69 |  |  | 1778 | 4.50 |  |
| 4 | surrogate consent | Yes | 3769 | 270 | 7.16 | <0.001 |  | 350 | 9.29 | <0.001 |
|  |  | No | 38047 | 508 | 1.34 |  |  | 1584 | 4.16 |  |
| 5 | Use of mobility aid | Yes | 5369 | 219 | 4.08 | <0.001 |  | 352 | 6.56 | <0.001 |
|  |  | No | 38456 | 586 | 1.52 |  |  | 1639 | 4.26 |  |
