# Supplementary material for: Nationwide Real‐World Modeling of Surgical Outcomes in Elderly Patients: Incorporating Geriatric‐Specific Risk Factors Into Prediction of Mortality and Morbidity
Source: Ann Gastroenterol Surg. 2026 Jan 11;10(3):904–19. doi: 10.1002/ags3.70164 (PMC13178288; doi:10.1002/ags3.70164)
Supplement: Supplementary file 4 — Table S4: Number of missing data by age and surgical procedure. [file AGS3-10-904-s003.docx]

| Supplementary table_4 Number of missing data by age and surgical procedure | | | | | | | | | | | | | | | | | | | |
| --- | --- | --- | --- | --- | --- | --- | --- | --- | --- | --- | --- | --- | --- | --- | --- | --- | --- | --- | --- |
|  | **fall history** | | |  | **Hospitalization from home** | | |  | **surrogate consent** | | |  | **Use of mobility aid** | | |  | **History of Dementia** | | |
|  | observed cases | missing | proportion of missing |  | observed cases | missing | proportion of missing |  | observed cases | missing | proportion of missing |  | observed cases | missing | proportion of missing |  | observed cases | missing | proportion of missing |
| Variables | 59528 | 5340 | 8.23% |  | 59092 | 5776 | 8.90% |  | 59730 | 5138 | 7.92% |  | 62578 | 2290 | 3.53% |  | 59843 | 5025 | 7.75% |
| Age |  |  |  |  |  |  |  |  |  |  |  |  |  |  |  |  |  |  |  |
| i) 65-69 | 10610 | 885 | 7.70% |  | 10530 | 965 | 8.39% |  | 10631 | 864 | 7.52% |  | 11068 | 427 | 3.71% |  | 10658 | 837 | 7.28% |
| ii) 70-74 | 17192 | 1512 | 8.08% |  | 17088 | 1616 | 8.64% |  | 17247 | 1457 | 7.79% |  | 18025 | 679 | 3.63% |  | 17266 | 1438 | 7.69% |
| iii) 75-79 | 14188 | 1301 | 8.40% |  | 14105 | 1384 | 8.94% |  | 14258 | 1231 | 7.95% |  | 14944 | 545 | 3.52% |  | 14280 | 1209 | 7.81% |
| iv) 80-84 | 10795 | 980 | 8.32% |  | 10688 | 1087 | 9.23% |  | 10823 | 952 | 8.08% |  | 11373 | 402 | 3.41% |  | 10844 | 931 | 7.91% |
| v) 85-89 | 5222 | 530 | 9.21% |  | 5170 | 582 | 10.12% |  | 5244 | 508 | 8.83% |  | 5559 | 193 | 3.36% |  | 5261 | 491 | 8.54% |
| vi) 90- | 1521 | 132 | 7.99% |  | 1511 | 142 | 8.59% |  | 1527 | 126 | 7.62% |  | 1609 | 44 | 2.66% |  | 1534 | 119 | 7.20% |
| procedure |  |  |  |  |  |  |  |  |  |  |  |  |  |  |  |  |  |  |  |
| DG | 16834 | 1649 | 8.92% |  | 16703 | 1780 | 9.63% |  | 16914 | 1569 | 8.49% |  | 17973 | 510 | 2.76% |  | 16939 | 1544 | 8.35% |
| LAR | 9801 | 801 | 7.56% |  | 9734 | 868 | 8.19% |  | 9822 | 780 | 7.36% |  | 10302 | 300 | 2.83% |  | 9848 | 754 | 7.11% |
| RHC | 13586 | 1105 | 7.52% |  | 13493 | 1198 | 8.15% |  | 13629 | 1062 | 7.23% |  | 14374 | 317 | 2.16% |  | 13667 | 1024 | 6.97% |
| TG | 5938 | 590 | 9.04% |  | 5891 | 637 | 9.76% |  | 5974 | 554 | 8.49% |  | 6345 | 183 | 2.80% |  | 5975 | 553 | 8.47% |
| PD | 6715 | 724 | 9.73% |  | 6658 | 781 | 10.50% |  | 6732 | 707 | 9.50% |  | 6789 | 650 | 8.74% |  | 6740 | 699 | 9.40% |
| ESO | 3082 | 197 | 6.01% |  | 3063 | 216 | 6.59% |  | 3076 | 203 | 6.19% |  | 3171 | 108 | 3.29% |  | 3087 | 192 | 5.86% |
| HEP | 3572 | 274 | 7.12% |  | 3550 | 296 | 7.70% |  | 3583 | 263 | 6.84% |  | 3624 | 222 | 5.77% |  | 3587 | 259 | 6.73% |
| abbreviations; LAR; low anterior resection, PD; pancreaticoduodenectomy, DG; distal gastrectomy, ESO; esophagectomy, HEP; hepatectomy, RHC; right-hemi colectomy, and TG; total gastrectomy | | | | | | | | | | | | | | | | | | | |
